# Task Feasibility Maximization Using Model-Free Policy Search and Model-Based Whole-Body Control

**DOI:** 10.3389/frobt.2020.00061

**Published:** 2020-06-04

**Authors:** Ryan Lober, Olivier Sigaud, Vincent Padois

**Affiliations:** ^1^Fuzzy Logic Robotics, Paris, France; ^2^Institut des Systèmes Intelligents et de Robotique, Sorbonne Université, CNRS UMR 7222, Paris, France; ^3^Auctus, Inria, Talence, France

**Keywords:** humanoids, reinforcement learning, policy Search, whole-body control, iCub humanoid robot

## Abstract

Producing feasible motions for highly redundant robots, such as humanoids, is a complicated and high-dimensional problem. Model-based whole-body control of such robots can generate complex dynamic behaviors through the simultaneous execution of multiple tasks. Unfortunately, tasks are generally planned without close consideration for the underlying controller being used, or the other tasks being executed, and are often infeasible when executed on the robot. Consequently, there is no guarantee that the motion will be accomplished. In this work, we develop a proof-of-concept optimization loop which automatically improves task feasibility using model-free policy search in conjunction with model-based whole-body control. This combination allows problems to be solved, which would be otherwise intractable using simply one or the other. Through experiments on both the simulated and real iCub humanoid robot, we show that by optimizing task feasibility, initially infeasible complex dynamic motions can be realized—specifically, a sit-to-stand transition. These experiments can be viewed in the accompanying Video S1.

## 1. Introduction

Highly redundant robots, such as humanoids, have enormous potential industrial and commercial utility. Unfortunately producing feasible and useful behaviors on real robots is a challenging undertaking, particularly when the robot must interact with the environment. This is caused, in large part, by the fact that there are always errors between what is planned, or simulated, and what is executed on a real robot due to modeling errors and perturbations. Consequently, an automatic method of resolving these errors on real platforms is necessary for robots to attain true autonomy. Model-based control alone cannot resolve these issues because the many possible causes could not be practically modeled for a general case (Mansard et al., 2018). Similarly, even the most sample efficient end-to-end learning methods (e.g., Gu et al., 2016) would also fail because training a model on a real robot would require an unrealistic number of evaluations, or rollouts. In this study, we show that by combining control and learning techniques, we can create low-dimensional high-level abstractions of whole-body behaviors and efficiently correct initially infeasible motions on real robots.

**Figure 1:**
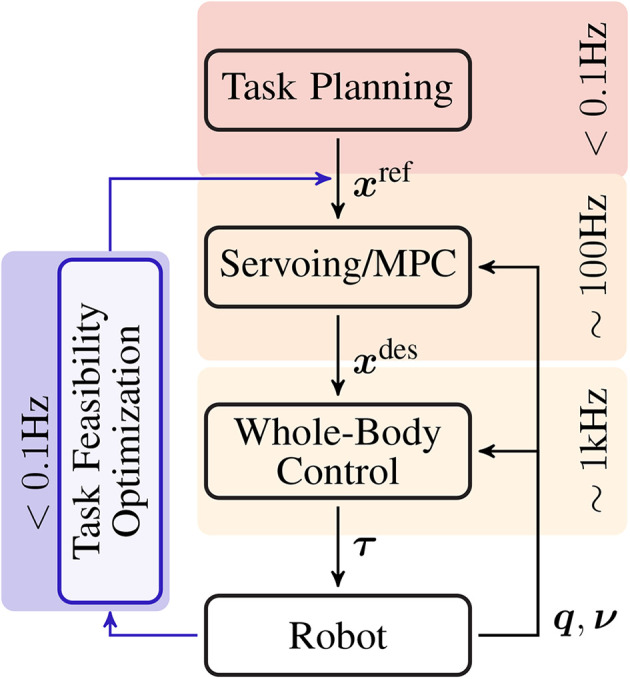
A modern control hierarchy for highly redundant robotic systems, e.g., humanoid robots. At the lowest level is whole-body control, which determines the torques needed to accomplish a set of tasks. At the intermediate level, these tasks are controlled by the servoing/MPC level where task trajectory errors are compensated using feedback. Finally the task trajectories are provided by high-level planning, which is usually a combination of operator expertise and automated planning. The task feasibility optimization loop proposed in this paper is designed to correct infeasible tasks produced by this architecture.

Modern control architectures employ multiple control levels in order to decouple complex behaviors into manageable control problems (Ibanez et al., 2017). At the lowest level is *reactive whole-body control*, where joints torques are calculated at high frequency (~1 kHz) given one or more tasks (Khatib et al., 2004). The control problem can be written as a constrained convex optimization, where the objective function is a combination of task errors, and the constraints are the equations of motion, articulation and actuation limits, and contacts (Bouyarmane and Kheddar, 2011; Salini et al., 2011; Saab et al., 2013). Task errors are dictated by desired task values which come from the next level of *task servoing*. At this level, closed loop controllers are used to servo task trajectories using state feedback (PID) and/or Model Predictive Control (MPC) schemes at frequencies between 100 and 10 Hz (Ibanez et al., 2014; Koenemann et al., 2015). These task trajectories generate the reference values, which are used by task servoing, and come from the higher-level *open-loop planning* which takes seconds to minutes, and generally combines operator expertise and automated planning algorithms (Bouyarmane and Kheddar, 2012; Pham, 2014). This control hierarchy of planning, servoing, and whole-body control is presented in Figure 1.

Because the control problem is abstracted in the task servoing and planning levels, there is no guarantee that the task trajectories will be executed properly by the lower control layers. Furthermore, tasks may conflict with one another and/or the system constraints (Bouyarmane and Kheddar, 2015; Wieber et al., 2017). The end result is typically unstable or undesirable whole-body behaviors, and we qualify these tasks as *infeasible*. Prioritization techniques are used to manage perturbations engendered by infeasible tasks at the whole-body control level, but are difficult to tune and only circumvent the problem. Moreover, tasks infeasibilities change over the course of the movement so applying static priorities may be overly restrictive (Lober et al., 2015; Modugno et al., 2016a). Likewise, tuning/scheduling the task servoing gains not only modifies the task trajectories, but also changes the controller's impedance, which may be undesirable for the application. Hence, decoupling the impedance problem from the trajectory shaping problem is not only prudent, but simplifies each because well-designed task trajectories should alleviate the need for priority and gain tuning.

Given that it is the task reference values which generate the infeasible control solutions, the task trajectories must be altered. To do so, the errors induced by infeasibilities can be measured and the task trajectories may be modified to reduce them. Additionally, the servoing and whole-body control levels with all of their parameters, as well as the robot's dynamics and environment, need to be taken into account. Given the complexity of these requirements, it is impractical to analytically model the relationship between task trajectories and feasibility. One solution is therefore to use model-free policy search (PS) techniques (Stulp and Sigaud, 2013) to modify the trajectories through trial and error by minimizing a cost function.

The objective of this study is to establish the task feasibility optimization loop, shown on the left in Figure 1, by iteratively improving task trajectories using PS and exploiting the model-based control layers. While task-level model-based whole-body control is the state of the art in terms of real-world humanoid robot control, hand-tuning trajectories is not realistic. Stochastic trajectory optimization is a great candidate for automating this process, but it suffers from the curse of dimensionality when applied to joint-space trajectory optimization. Our approach attempts to reconcile these techniques in a simple and pragmatic task-level learning framework. Building on the work in Lober et al. (2016), we first formalize the relationship between task trajectories and parameterized policies in the whole-body control architecture. We then develop a task feasibility cost, the penalty function, from simple principles which measure the infeasibility of a task. This feasibility cost is then minimized. In robotics, it is advantageous, from both a time and monetary standpoint, to perform PS with the fewest possible rollouts. To this end, we use Bayesian Optimization (BO) for its sample efficiency. BO solvers usually require fewer trials to obtain an optimal solution and have become a popular choice in robotics because of this efficacy (Calandra et al., 2014; Cully et al., 2015; Antonova et al., 2016; Englert and Toussaint, 2016).

**Figure 2:**
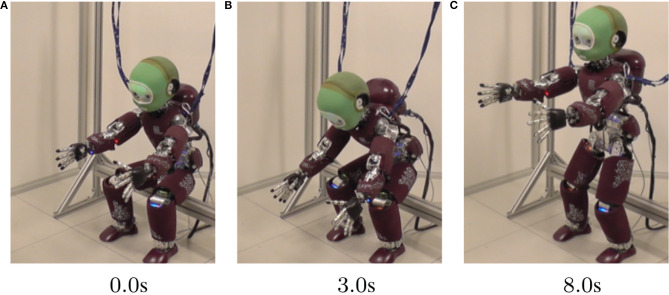
In **(A,B,C)**, we show a time-lapse of a feasibility-optimized standing motion executed on an iCub robot.

To study task feasibility optimization, we explore the dynamically challenging activity of moving from sitting to standing for the humanoid robot iCub, both in simulation and on the real robot. This motion requires contact switching and potentially unstable dynamic equilibrium to succeed. In addition to a postural impedance task, a Center of Mass (CoM) task is used to manage the sit-to-stand transition. The trajectory of the CoM task is optimized to minimize the task feasibility cost. Through these experiments, we demonstrate that by combining analytical model-based controllers with data-driven model-free PS techniques, we are able to solve problems which would be otherwise intractable using simply one or the other—e.g., producing feasible dynamically complex motions on real robots, like the example shown in Figure 2.

In this paper, we demonstrate for the first time that a feasibility maximization technique can be applied to a real humanoid robot and result in significantly improved whole body trajectories. More precisely, our contributions are the following:

- We define a task feasibility cost function which, when optimized over a set of concurrent tasks, results in improved trajectory from the robot.
- We show that this task feasibility cost function can be optimized in very few iterations using a Bayesian Optimization technique where the minimization of the acquisition function is performed using the CMA-ES algorithm.
- We evaluate this method in simulation and on the real iCub humanoid robot and show through a simple proof-of-concept experiment that it can result in significant improvement of the generated trajectories in a way that is practical for real world learning.

## 2. Methods

In this section, we describe the methods and tools used to develop task feasibility optimization. We begin with an overview of the underlying whole-body control architecture and conclude with a description of PS. Here the policy to be optimized is parameterized by the CoM task trajectory.

### 2.1. Control Architecture

Model-based whole-body controllers determine at each control instant, *k*, the joint torques, ***τ***(*k*), necessary to accomplish some set of *n*_*T*_ tasks, for all of the degrees of freedom of the given robot, while respecting physical constraints such as the equations of motion, articulation and actuation limits, and contacts. These controllers can be formulated using analytical null-space projection methods (Dietrich et al., 2015), or multicriterion convex optimization problems using weighted (Salini et al., 2011; Saab et al., 2013) and/or hierarchical objective scalarization (Escande et al., 2014). Regardless of the formalism, any of these controllers can be abstracted to the following generic function,

(1) $$\begin{array}{l} \tau \left(k\right)=\text{controller }\left(s\left(k\right),C\left(k\right),T_{i}\left(k\right)\right)\;\forall i\in \left\{1,2,\ldots ,n_{T}\right\}\;, \end{array}$$

which takes the robot's state, ***s***(*k*), its constraints, C(k), and some tasks *T*_*i*_(*k*), as inputs and outputs the joint torques. The robot state, contains ***q***(*k*), the generalized coordinates, and ***ν***(*k*), the generalized velocities. The variable C(k) contains any active constraints, e.g., joint and actuator limits, contacts, etc. Tasks may be described in any number of ways in either operational-space or joint-space, but all are governed by desired task values provided by task servoing.

In an earlier version of this method, presented in Lober et al. (2016), the whole-body controller described in Salini et al. (2011) is used. In this work, the whole-body control algorithm used is the momentum-based hierarchical controller developed in Nava et al. (2016) and Pucci et al. (2016), which has momentum tracking, *T*_m_, and joint impedance tasks, *T*_j_—the most important of which is the former. Equation (1) can then be written,

(2) $$\begin{array}{l} \tau \left(k\right)=\text{controller }\left(s\left(k\right),C\left(k\right),T_{\text{m}},T_{\text{j}}\right)\text{.} \end{array}$$

For the momentum task, the desired value is entirely determined by the desired CoM acceleration, ${\ddot{x}}_{\text{CoM}}^{\text{des}}$, and is provided by a proportional-integral servoing controller,

(3) $$\begin{array}{l} {\ddot{x}}_{\text{CoM}}^{\text{des}}={\ddot{x}}_{\text{CoM}}^{\text{ref}}-K_{p}\left({\overset{\cdot }{x}}_{\text{CoM}}-{\overset{\cdot }{x}}_{\text{CoM}}^{\text{ref}}\right)-K_{i}\left(x_{\text{CoM}}-x_{\text{CoM}}^{\text{ref}}\right)\;, \end{array}$$

where *K*_*p*_ and *K*_*i*_ are the proportional and integral gain matrices, respectively. The CoM reference values, $x_{\text{CoM}}^{\text{ref}}$, ${\overset{\cdot }{x}}_{\text{CoM}}^{\text{ref}}$, and ${\ddot{x}}_{\text{CoM}}^{\text{ref}}$ are provided by a CoM trajectory. The choice of this reference is crucial for a successful whole-body motion.

In the context of the sit-to-stand example explored here, a finite-state-machine (FSM) composed of two states, coordinates the standing motion in the controller. In the “Sit” state, the robot is seated on the bench, and the two contacts at the left and right upper legs are controlled to keep the equilibrium. When a resultant ground reaction force greater than 150 N is detected, the FSM switches to the “Stand” state, moving the bench contacts to the left and right heels in the whole-body controller.

### 2.2. Proposed Approach for Policy Search

Policy search methods are black-box optimization techniques for iteratively learning control policies rather than programming them by hand (Deisenroth et al., 2013). Model-free parameterized PS lends itself to robotics as it precludes the need for an analytical transition dynamics model and allows high-dimensional problems to be handled with few parameters. In keeping with reinforcement learning nomenclature, we define the agent of this system, the humanoid robot (iCub), and its discrete-time states are ***s***(*k*). The actions of the agent, ***a***(*k*), are then the actuator torques, developed at each control instant, ***a***(*k*) = ***τ***(*k*). The control policies, ***π***(***a***(*k*)|***s***(*k*)), determine the action at time *k* given the current state. The policies are mappings from task reference inputs, $x_{i}^{\text{ref}}$, ${\overset{\cdot }{x}}_{i}^{\text{ref}}$, and ${\ddot{x}}_{i}^{\text{ref}}\;\forall i\in \left\{1,2,\ldots ,n_{T}\right\}$, to ***τ***, using the whole-body reactive controller described in section 2.1. It should be noted that this mapping is not bijective and cannot be described by a differentiable function. In order to modify and improve ***π***(*k*), three levers can be used. The first one is related to the modification of the parameters defining the tasks achievement dynamics, i.e., the PID gains. The second one is related to the relative weight of each task in the QP controller. While both approaches can yield modifications of the robot's resulting motion (Buchli et al., 2011; Modugno et al., 2016b), they do not lead to an explicit improvement of the definition of the trajectory to be tracked. In a long-term learning perspective it may actually be more beneficial to improve trajectories directly in order to render them feasible with respect to the physical constraints of the robot and compatible one with another. So, assuming fixed whole-body controller parameters (PID gains, task weights), the mapping depends only on ***s***(*k*) and the task control objectives at each time step. Therefore, in order to modify ***π***(*k*) we must modify the task reference values, i.e., the task trajectories.

### 2.3. Policy Parameterization: Task Trajectories

Given the high dimensionality of the system's states and actions (iCub possesses 32 main articulation yielding *dim*(***s***(*k*)) = (32+6) × 2 and *dim*(***a***(*k*)) = 32), we opt for a parameterized policy representation. As presented in section 2.2, task trajectories uniquely determine the evolution of the system, and therefore provide a condensed representation of ***π*** for a given motion. The task trajectories, and hence ***π***, are parameterized by a series of keyframes/waypoints, which represent task coordinates of particular importance. A single position waypoint is given by ***θ***_*i*_, while a set of *n*_θ_ waypoints is denoted Θ = [***θ***_1_***θ***_2_ … ***θ***_*n*θ_]. From Θ, a policy must be formed using a parameterized function, **π**_*θ*_ = ***ρ***(Θ), where the ***ρ***(Θ) function can be chosen from a variety of parameterized trajectory generators: e.g., splines, polynomials, optimal control methods, etc. Here, we use the formulation proposed by Kunz and Stilman (2012), which produces a time-optimal trajectory through Θ, with a duration, *t*_***π***_, dependent on the velocity and acceleration limits imposed on the movement. For this study, we focus on the CoM task trajectory, which will guide the robot from a seated state to a standing state and therefore write the policy as, ***π*** = ***ρ***(Θ^CoM^), where Θ^CoM^ are the CoM waypoints. Note that any task trajectories can be used in the parameterization of ***π***.

Because of the nature of the standing motion studied here, we may further restrict the parameterization. Since the robot starts in a seated posture and finishes in a standing posture, the initial, ***θ***_start_, and final, ***θ***_end_ = ***θ***_*n*_θ__, waypoints of the movement remain constant. As such, only the intermediate waypoints are used to modify **π**_*θ*_. Here, we consider only one intermediate CoM waypoint, ***θ***_mid_, simplifying the policy parameterization to,

(4) $$\begin{array}{l} \pi_{\theta }=\rho \left(\theta_{\text{mid}}\right)\;. \end{array}$$

### 2.4. Policy Rollouts: Task-Set Execution

Given a parameterized policy, **π**_*θ*_, we wish to determine the evolution of the robot's states and actions. The policy is therefore rolled-out, meaning that the task-set is executed on the robot, either in simulation or reality, and the state and action data are recorded,

(5) $$\begin{array}{l} \left\{S,A\right\}=\text{rollout}\left(\pi_{\theta }\right)\;, \end{array}$$

where S and A are the concatenations of the states and actions over the entire rollout. This implies that the full control architecture, as described in section 2.1, is employed until the task execution is complete, meaning that the execution must occur in a finite amount of time and should be finished in the duration dictated by the CoM policy ***ρ***(Θ^CoM^), $t_{\pi }^{\text{CoM}}$. However, if the robot falls, then ***π***^CoM^ will not be completed in $t_{\pi }^{\text{CoM}}$. The policy rollouts are therefore assigned a maximum execution time, $t_{\text{max}}>t_{\pi }^{\text{CoM}}$, to allow for possible delays in the task execution but to avoid recording failed rollouts indefinitely. Here, we arbitrarily select $t_{\text{max}}=1.5\times t_{\pi }^{\text{CoM}}$.

### 2.5. Penalty Function: Task Feasibility Cost

In order to evaluate the policy rollouts, we use a penalty function based on three component cost functions, which evaluate the performance of the policy and are based on generic optimal control principles. These costs are calculated a posteriori on the rollout data determined in (5).

Using the state information S, we can determine how the CoM evolved over the course of a single rollout. We first examine how well the CoM position, ***x***_CoM_(*k*), tracked the references, $x_{\text{CoM}}^{\text{ref}}\left(k\right)$, provided by **π**_*θ*_, during the rollout and develop the *tracking cost*,

(6) $$\begin{array}{l} j_{t}=\overset{N}{\underset{k=0}{\sum }}\|x_{\text{CoM}}\left(k\right)-x_{\text{CoM}}^{\text{ref}}\left(k\right){\|}_{2}^{2}\;, \end{array}$$

where *N* is the total number of time steps. We define the actual total duration of the rollout, *t*_end_ = *NΔt*, where Δ*t* is the control sampling period, and $t_{\pi }^{\text{CoM}}\leq t_{\text{end}}\leq t_{\text{max}}$. If a task error is perfectly minimized by the controller, then it goes to zero, meaning that the robot perfectly executes **π**_*θ*_. Any error in the position tracking then reflects imperfect optimization and consequently a task infeasibility associated with the current policy. We assume that the ultimate objective of the standing motion, and any point-to-point trajectory for that matter, is to reach its final waypoint. With this in mind a *goal cost* is developed,

(7) $$\begin{array}{l} j_{g}=\overset{N}{\underset{k=0}{\sum }}\frac{k\Delta t}{t_{\pi }}\|x_{\text{CoM}}\left(k\right)-\theta_{\text{end}}{\|}^{2}\;, \end{array}$$

where ***x***_CoM_(*k*) − ***θ***_end_ is the difference between the CoM task position at time step *k* and the final waypoint in its trajectory. The weight of this difference increases linearly from zero with time. This means that the distance to the goal waypoint becomes more important as time elapses. Finally, we wish to determine the most energetically optimal motion, by minimizing the actions, ***a*** (i.e., the control inputs, ***τ***) using an *energy cost*,

(8) $$\begin{array}{l} j_{e}=\beta \overset{N}{\underset{k=0}{\sum }}\|\tau \left(k\right){\|}^{2}\;. \end{array}$$

Energy cannot be directly compared with Cartesian distances, so the β parameter must be introduced to scale *j*_*e*_ for meaningful comparison with *j*_*t*_ and *j*_*g*_. Here, we use β = 1.0e−4. The penalty function, or *feasibility cost* can be calculated by summing the component costs, and averaging over *t*_end_ to account for rollouts with different timescales,

(9) $$\begin{array}{l} j_{f}=\text{penalty }\left(\left\{S,A\right\}\right)=\frac{j_{e}+j_{t}+j_{g}}{t_{\text{end}}}\;. \end{array}$$

With (10) we can estimate the feasibility of **π**_*θ*_. However, this estimate has no absolute significance on its own. There is no threshold value for determining analytically if **π**_*θ*_ was successful in a high-level sense (i.e., the robot stood up). Given this ambiguity, we take the $j_{f}^{0}$ of the initial $\pi_{\theta }^{0}$ as the reference with which all other $\pi_{\theta }^{i}$ are compared using, ${\hat{j}}_{f}^{i}=\frac{j_{f}^{i}}{j_{f}^{0}}$, where *i* indicates the rollout number. This means that the initial, $\pi_{\theta }^{0}$, has a feasibility cost equal to 1.0 and any $\pi_{\theta }^{i}$ which produces a ${\hat{j}}_{f}^{i}<1.0$ represents an improvement in task feasibility, and vice versa for ${\hat{j}}_{f}^{i}>1.0$.

While defined with respect to the CoM task, these costs are applicable to any other form of control task and provide general feasibility indicators: a task which cannot be achieved either in terms of tracking or in terms of target reaching or which achievement requires very high energy is hardly or not feasible. Model-based metrics can be used to define the general notion of feasability (Lober, 2017, chap. 3,6; Lober, 2017, chap. 7) actually shows that their is a strong positive correlation between these model-based metrics and the ones used in this work. This correlation is not further explored in this proof-of-concept article.

### 2.6. Optimizing the Policies: Bayesian Optimization

Since the transition dynamics, P(s(k+1)|s(k),a(k)), are governed by the equations of motion with changing contacts, P is a discontinuous and time-varying non-linear function. Therefore, in order to optimize the policy parameters given a scalar reward or penalty, non-convex black-box solvers must be used. The downside to these solvers is that they typically require many rollouts (parameter, $\theta_{\text{mid}}^{i}$, and cost, ${\hat{j}}_{f}^{i}$, samples) to converge on a local optimum. In humanoid robotics, rollouts are time consuming and dangerous. As a consequence, sample efficiency is of the highest importance in PS. This, in addition to the low dimensionality of the parameter space, permits the use of BO to optimize, ***θ***_mid_. BO derives its sample efficiency from explicitly modeling the latent parameter to cost mapping using Gaussian Processes (GP), and then using this model, or surrogate function, to explore the parameter space. The actual minimization is performed on an acquisition function which combines the cost means and variances provided by the GP to balance exploitation with exploration (Brochu et al., 2010). In this study, the Lower Confidence Bound (LCB) acquisition function is used (see Cox and John, 1992) and minimized with a Covariance Matrix Adaptation Evolutionary Strategy solver (see Hansen, 2006). The parameter search space is bounded using box constraints around a 3-dimensional cube of possible $\theta_{\text{mid}}^{i}$, positions as shown in **Figure 4A**. The incumbent solution is taken as the best parameter and cost observation from the rollouts, $\theta_{\text{mid}}^{*}$ and $j_{f}^{*}$; therefore, the optimization does not depend on the sequence in which the rollouts are performed. One drawback to BO is that it does not guarantee convergence in most cases (a comparison with other optimization approaches can be found in Lober, 2017, chap. 9,10). Here, convergence is assumed when BO proposes a new $\theta_{\text{mid}}^{i}$ which satisfies,

(10) $$\begin{array}{l} \|\theta_{\text{mid}}^{i}-\theta_{\text{mid}}^{*}\|\leq \gamma \;, \end{array}$$

where γ is a distance threshold, or the number of iterations has exceeded some maximum value.

### 2.7. Task Feasibility Optimization

Finally, the task feasibility optimization loop can be written as shown in Algorithm 1. Starting from policy parameters $\theta_{\text{mid}}^{i}=\theta_{\text{mid}}^{0}$, $\pi_{\theta }^{i}$ is generated using (4), and rolled out on either the simulated or real robot. The resulting states and actions are used to calculate a feasibility cost with (10), which is subsequently scaled. The GP of the BO surrogate function is then trained with the new parameter and cost data, $\left\{\theta_{\text{mid}}^{i},{\hat{j}}_{f}^{i}\right\}$, and the next $\theta_{\text{mid}}^{i}$ is determined by minimizing the LCB acquisition function. The new $\theta_{\text{mid}}^{i}$ is then compared to the incumbent solution $\theta_{\text{mid}}^{*}$ to determine if convergence has been achieved. If so then the incumbent is returned.

**Algorithm 1:** Task Feasibility Optimization

1:	Given initial policy parameters: $\theta_{\text{mid}}^{i}=\theta_{\text{mid}}^{0}$.	
2:	**do**	
3:	$\pi_{\theta }^{i}=\rho \left(\theta_{\text{mid}}^{i}\right)$	⊳ generate policy from parameters
4:	${\left\{S,A\right\}}^{i}=\text{rollout}\left(\pi_{\theta }^{i}\right)$	⊳ rollout the policy
5:	$j_{f}^{i}=\text{penalty}\left({\left\{S,A\right\}}^{i}\right)$	⊳ calculate the feasibility cost
6:	${\hat{j}}_{f}^{i}=\frac{j_{f}^{i}}{j_{f}^{0}}$	⊳ scale the cost
7:	GP.Train$\left(\left\{\theta_{\text{mid}}^{i},{\hat{j}}_{f}^{i}\right\}\right)$	⊳ train the BO surrogate function
8:	$\theta_{\text{mid}}^{*}=\text{arg min}\;\left\{{\hat{j}}_{f}^{1},{\hat{j}}_{f}^{2},\ldots {\hat{j}}_{f}^{i}\right\}$	⊳ get incumbent solution
9:	$\theta_{\text{mid}}^{i}=\text{arg min}$ LCB	⊳ minimize acquisition function
10:	**while** (11) ≠ True and *i* < Max Iter.	⊳ convergence criteria
11:	**return** $\theta_{\text{mid}}^{*}$	⊳ return incumbent solution

## 3. Experiments

The task feasibility optimization is tested using a dynamically complex scenario in which the iCub robot (Metta et al., 2008) starts from a seated position on a stationary bench and must transition to standing. The bench contacts are 22cm from the ground and on the back of the iCub's upper thigh links. The toes are in contact with the ground. The initial posture is chosen to ensure that the ground-plan (*x*-*y*) projection of starting CoM position is within the Polygon of Support (PoS) defined by the bench and ground contact locations. The contacts are managed by the FSM described in section 2.1. The initial policy parameters, $\theta_{\text{mid}}^{0}$, are chosen between ***θ***_start_ and ***θ***_end_, resulting in a straight line CoM trajectory. A full execution of the whole-body controller constitutes a single policy rollout. The rollout is completed when the robot reaches ***θ***_end_ to within 3.0 cm of accuracy, or if *t*_end_ > *t*_max_.

**Figure 3:**
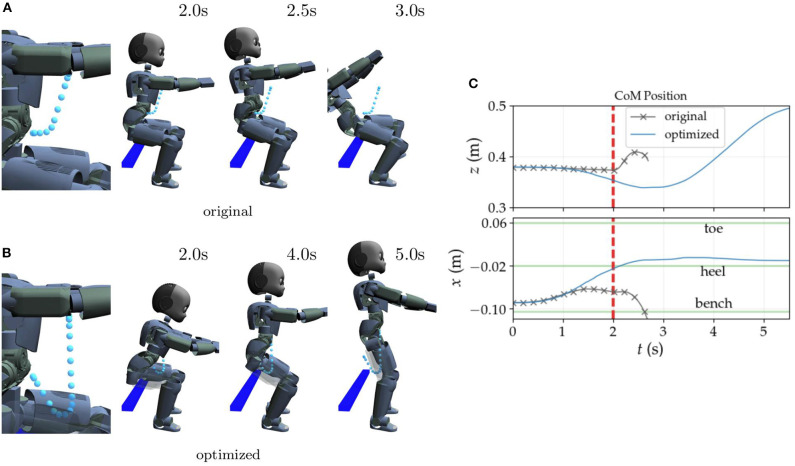
Example of original and optimized CoM reference trajectories and their resultant whole-body motions. The original policy **(A)** produces an unstable standing motion causing the robot to lose balance. The optimized policy, however, produces a successful sit-to-stand transition. The right hip is translucent in **(B)** to make the reference trajectory visible. **(C)** Shows the evolution of the CoM for the original and optimized policies. The original CoM curves are cut off after 2.7 s when the robot loses balance. The red dashed line indicates the moment when the bench contacts are deactivated in the whole-body controller.

The rollouts are first carried out in simulation using Gazebo as the simulation environment with the ODE physics engine. Figure 3 exemplifies through snapshots typical results of CoM trajectory optimization with roll-outs in simulation only and using the whole-body controller described in Salini et al. (2011).

PS is iterated until one of the convergence criteria detailed in section 2.6 is met. In this study γ = 1.0 cm, and the maximum number of iterations is 30 in simulation and 10 on the real robot. The optimal policy parameters, $\theta_{\text{mid}}^{*}$ are then used to generate $\pi_{\theta }^{*}$ which is rolled out on the real iCub. This rollout is used to demonstrate that task feasibility can be initially optimized in simulation and produce feasible motions on the real robot. With the $\pi_{\theta }^{*}$ from simulation as a starting point, the PS is continued by performing rollouts on the real iCub. For these rollouts we look at two cases. In the first, the BO surrogate function training is *bootstrapped* with training data from the simulated rollouts and further trained on data from the real rollouts. In the second *non-bootstrapped* case, the surrogate function is trained only using the real rollout data. For both cases, the $\pi_{\theta }^{*}$ from the simulation rollouts is used as the initial policy for the real rollouts, warm starting the PS. To limit the number of falls, the BO search space bounds are restricted to a 10cm cube around the initial $\theta_{\text{mid}}^{*}$, for the real rollouts. Ten rollouts are performed for both cases. All code and data for these experiments is open-source and can be found here: https://github.com/rlober/task-optim. Please see the accompanying Video S1 for a detailed look at the rollouts.

## 4. Results

**Figure 4:**
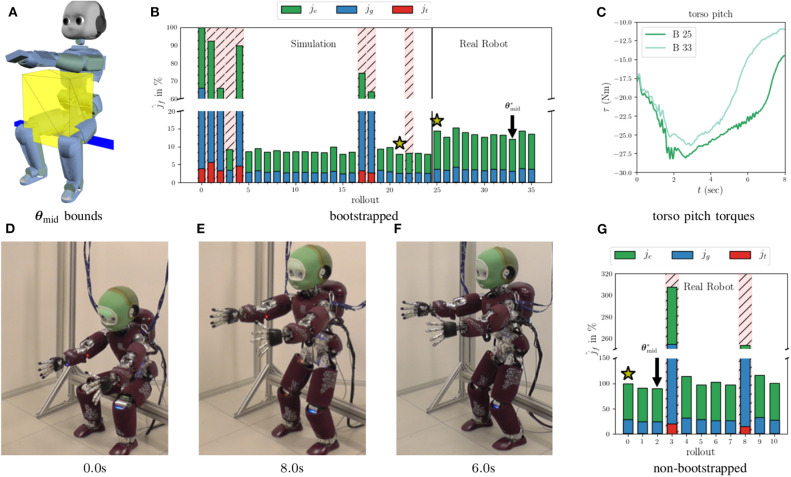
**(A)** Shows the bounds initially used for the BO in simulation. For the real rollouts, these bounds are then further restricted to a 10 cm cube around the initial ***θ***_mid_. **(B)** Shows the feasibility cost percentages (bootstrapped case) from the rollouts in both simulation and on the real robot. **(C)** Shows the evolution of the torso pitch joint torques for the rollouts 25 and 33 in the bootstrapped case. The rollouts which produced a failure (falling) are indicated by the red hatched backgrounds. The optimal (best observed costs) policy parameters, $\theta_{\text{mid}}^{*}$, are indicated for both real rollout cases. **(G)** Shows the costs for the non-bootstrapped case. **(D)** Shows the initial posture of the iCub robot. **(E,F)** Show the final standing posture of the optimized motions for the bootstrapped and non-bootstrapped cases, respectively.

**Figure 5:**
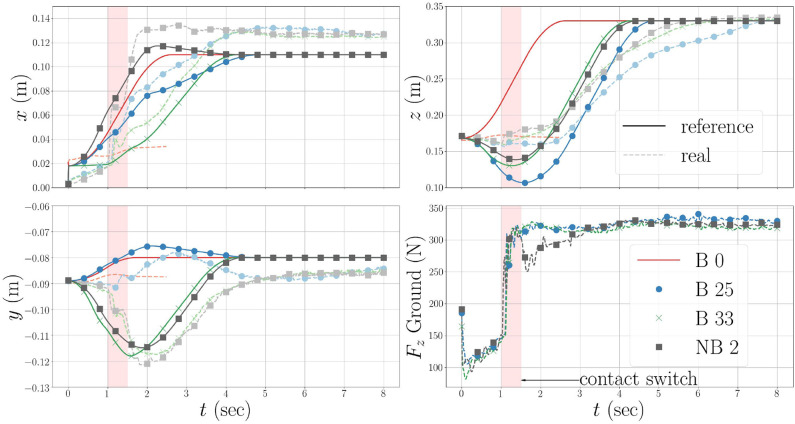
The evolution of the CoM trajectories generated by the original and optimized policies. “B” indicates the bootstrapped case, and “NB” the non-bootstrapped case. B 0 is the original policy executed in simulation. The optimal policy found in the simulated rollouts comes from B 21, or the 21st rollout of the bootstrapped case. B 25 and NB 0, i.e., the first real rollouts for the bootstapped and non-bootstrapped cases, use the B 21 policy. This policy is indicated by the yellow stars in the cost curves in Figures 4B,G. B 33 is the optimal policy found during the real bootstrapped rollouts. NB 2 is the optimal policy found during the real non-bootstrapped rollouts. The solid lines are the reference values generated by **π**_*θ*_ and the lighter dashed lines are the real measured values. The original, B 0, real lines are cut off after 2.5s when the robot falls. The noisy B 0 force profile is omitted from the force plot, to not obfuscate the other force profiles.

In **Figure 5**, we see the evolution of the CoM for the original policy, B 0, and the policies optimized in simulation, B 25, the bootstrapped case, B 33, and the non-bootstrapped case, NB 2. The initial straight line CoM trajectory produces an unstable whole-body motion, which causes the robot to lose balance. The failing (i.e., falling) rollouts are indicated by the hatched red backgrounds in Figures 4B,G. Because the initial policy fails, the measured CoM position values for B 0 are not shown after 2.5 s due to noise, and the *F*_*z*_ values are omitted completely for clarity. After 24 rollouts in simulation (see Figure 4B), the task feasibility optimization converges to a policy which produces a successful sit-to-stand transition in both simulation and on the real robot. The rollouts can be watched in the accompanying Video S1. This policy comes from the rollout 21 in simulation, and is used as the policy for the initial real rollouts in both the bootstrapped and non-bootstrapped cases, B 25 and NB 0, respectively. This is confirmed by the real and reference CoM trajectories for B 25 in Figure 5. Had the motion failed, the real values would not have tracked the reference values as is the case for B 0.

Looking at the *z*-axis and *F*_*z*_ plots in Figure 5, we see that the optimal strategy, found in B 21, is to move the CoM downwards initially to increase the ground reaction force, and shift the robot's weight to the feet. This shift must come early in the execution of the CoM trajectory in order to achieve a contact switch in the FSM, and thus allow the CoM to continue tracking the trajectory references. When this policy is executed on the real robot in B 25 and NB 0, the results are successful, but higher *j*_*f*_, than predicted by simulation, are observed for both cases. These discrepancies come as no surprise, but indicate that some unpredicted factors come into play on the real robot and must therefore be accounted for.

Looking at NB 2 and NB 3, we have an example of an optimal policy and a costly policy which produces a fall. In these two rollouts, the policy parameters being tested are $\theta_{\text{mid}}^{*}\;=\;\theta_{\text{mid}}^{2}\;=\;{\left[\begin{array}{ccc} 0.12 & -0.124 & 0.115 \end{array}\right]}^{⊤}$and $\theta_{\text{mid}}^{3}={\left[\begin{array}{ccc} 0.12 & -0.02 & 0.115 \end{array}\right]}^{⊤}$, respectively.These parameters differ by only 10cm in the *y*-axis, which in theory, should not affect a sagittal plane motion. However, this subtle change in the trajectory makes the difference between optimality and catastrophic failure. We can see in the *y*-axis plot of Figure 5 that the optimal policies found both with and without bootstrapping possess this *y*-axis motion, contrary to the policy optimized in simulation, and clearly attempt to compensate for un-modeled infeasibilities in the real system. Given the sensitive nature of the sit-to-stand motion, hand-tuning the trajectory parameters would be a difficult chore even for an expert.

Figures 4B,G show the component costs for each rollout with and without bootstrapping. The percentage improvement, ${\hat{j}}_{f}^{i}\times 100$, of each cost shows how PS improves the motion with respect to the initial policy. The overall evolution of the total feasibility costs shows the almost binary nature of the sit-to-stand scenario—either the robot stands or it falls. Given this, and the nature of the BO used here, we do not observe smooth convergence. Furthermore, in both the bootstrapped and non-bootstrapped cases the convergence criterion from (11) is not attained. Nevertheless, the initial policies are improved using task feasibility optimization. The majority of this improvement arises thanks to a decrease in energy consumption. The energy savings come primarily from the large sagittally actuated pitch joints, and most notably that of the torso pitch. In Figure 4C, we see the torques from B 25 and B 33. Both policies produce a successful sit-to-stand motion, but the optimized policy solicits this actuator less than the initial policy and reaps large gains in the energy cost. As expected, the rollouts without bootstrapping show more aggressive exploration, with two policy failures at NB 3 and NB 8, than the rollouts with bootstrapping. This comes from the higher variance associated with the un-explored regions of the policy parameter search space. The exploration however, leads to an optimized motion which moves more quickly from the starting seated posture (see Figure 4D) to a standing posture, as shown by the trajectory in Figure 5, allowing it to spend less time in configurations which require large torques, than the solution found using bootstrapping. The decreased goal costs come from the fact that the robot is already standing after only 6.0 s (see Figure 4F) rather than 8.0s as is the case with the less aggressive movement found by the bootstrapped optimization (see Figure 4E). Around the solution space of feasible sit-to-stand CoM trajectories, the tracking cost has little impact on the total cost, but becomes more prominent when the policy fails.

## 5. Conclusion

The main takeaway from this work is that by exploiting an underlying model-based control architecture, we are able to abstract the problem of producing feasible motions to only a few task-space variables, which can affect drastic changes in the overall behavior. Given the low-dimensionality of the variables, PS can be applied in a sample efficient manner, making it viable for real robots which must learn quickly and efficiently with minimal failures (e.g., humanoids). This result should not be understated because motions planned in simulation, or using approximate models, are never executed perfectly on the real robot, and the infeasibilities must be corrected or tuned in most cases. Making this correction automatic, is a crucial step toward truly autonomous robots, and cannot practically be achieved on a real system with model-based control (Koenemann et al., 2015) or learning (Gu et al., 2016) alone. Our generic model-free approach allows any underlying whole-body controller to be used, as shown here and in Lober et al. (2016), and requires only the existence of task trajectories with which to optimize policies. Through the example sit-to-stand scenario, we show that task feasibility optimization provides an efficient interface between control and learning, which can resolve task infeasibilities and produce viable whole-body motions in both simulation and reality. In future work, it would be interesting to find automated ways of determining the policy parameters which need to be optimized, rather than having to specify them by hand. An advancement such as this would render task feasibility optimization entirely self-sufficient.

## Data Availability Statement

The datasets generated for this study are available on request to the corresponding author.

## Author Contributions

RL, OS, and VP developed the proposed control approach and wrote the final version of the paper. RL wrote the first draft of the paper, coded the algorithms, and ran the experiments.

## Conflict of Interest

The authors declare that the research was conducted in the absence of any commercial or financial relationships that could be construed as a potential conflict of interest.

## Supplementary Material

Video S1 This video summarizes the proposed method for task feasibility maximization and provides a view of some of the rollouts performed in simulation and on the real robot. It also provides a visual comparison of the optimized trajectories on the real robot in the cases of policy “optimized in simulation”, “optimized with bootstrapping” and “optimized without bootstrapping.”
